# Single-Use Surgilube® Packets as an Alternative to Ultrasound Gel Bottles

**DOI:** 10.7759/cureus.39666

**Published:** 2023-05-29

**Authors:** Joshua Davidson, Tanushree Bhatt, Deny Ponnachan, Hamza El Falah

**Affiliations:** 1 Pulmonary and Critical Care Medicine, BronxCare Health System, Bronx, USA; 2 Internal Medicine, BronxCare Health System, Bronx, USA

**Keywords:** burkholderia, surgilube, ultrasound gel, pocus (point of care ultrasound), hospital- acquired infection

## Abstract

Point-of-care ultrasound is widely used in hospitalized patients. Hospital-acquired infections attributed to contaminated multiuse ultrasound gel bottles have been increasingly reported, including *Burkholderia*, *Pseudomonas*, and *Acinetobacter* species. Surgilube’s chemical properties and sterile single-use packaging make it an appealing alternative to multi-use ultrasound gel bottles.

## Editorial

Ultrasonography is a radiation-free diagnostic test that utilizes high-frequency sound waves to generate an image of structures within the body. It has become a widely accepted bedside tool for assisting in rapid diagnosis and interventions [1]. The ultrasonography exam often requires a coupling media to displace air (which poorly transmits ultrasound waves) and fill the contours between tissue and the ultrasound probe. Coupling media reduces total acoustic impedance, which is the resistance an ultrasound beam encounters as it passes through a medium. The difference between the acoustic impedance of two materials (acoustic impedance mismatch) drives the soundwave’s reflection. The acoustic impedance of air is small relative to most biological tissue. The World Health Organization manual of diagnostic ultrasound recommends water-soluble gels for coupling media and gives an example formula composed predominantly of propylene glycol and a carbomer (an acrylic acid polymer); oil-based coupling media may dissolve rubber or plastic components of the transducers and are not recommended [2].

Recently, there have been reports of hospital-acquired infections attributed to contaminated ultrasound gel bottles in many countries, including the United States [3]. The most commonly reported organisms in the ultrasound-gel-associated hospital outbreaks are *Burkholderia* species, though other organisms have also been identified, including *Pseudomonas* and *Acinetobacter* species. Single-use ultrasound gel packets may offer a solution to this problem but may be expensive. Other coupling media options have been explored as a low-cost alternative to traditional ultrasound gels, including homemade formulas containing cornstarch, a carbohydrate, without loss of image integrity [4]. Surgilube® (HR Pharmaceuticals Inc, York, Pennsylvania) is a sterile chlorhexidine-containing, latex-free, water-soluble translucent gel consisting of predominantly propylene glycol and hypromellose (a synthetic polymer similar to cellulose) in a proprietary ratio designed as a medical lubricant [5]. It is traditionally used as a lubricant to minimize friction and, thus, patient discomfort during medical exams such as rectal and vaginal exams and facilitate the entry of medical devices such as catheters, endoscopes, and other surgical instruments into the body. Based on its primary components and the authors’ clinical experience, Surgilube® offers a viable alternative to multi-use ultrasound gels. As it is commonly dispensed in single-use packets and is widely available in many hospitals, single-use Surgilube® packets may offer a solution to contaminated multi-use ultrasound gel bottles and warrant further study.
